# Direct Prediction of the Toxic Gas Diffusion Rule in a Real Environment Based on LSTM

**DOI:** 10.3390/ijerph16122133

**Published:** 2019-06-17

**Authors:** Fei Qian, Li Chen, Jun Li, Chao Ding, Xianfu Chen, Jian Wang

**Affiliations:** 1 Department of Electronic Science and Technology, University of Science and Technology of China, Hefei 230029, China; fei123@mail.ustc.edu.cn (F.Q.); chenli18@mail.ustc.edu.cn (L.C.); lijun006@mail.ustc.edu.cn (J.L.); 2 State Key Laboratory of Fire Science, University of Science and Technology of China, Hefei 230029, China; dc707@ustc.edu.cn

**Keywords:** toxic gas, diffusion prediction models, deep learning algorithms, LSTM

## Abstract

Predicting the diffusion rule of toxic gas plays a distinctly important role in emergency capability assessment and rescue work. Among diffusion prediction models, the traditional artificial neural network has exhibited excellent performance not only in prediction accuracy but also in calculation time. Nevertheless, with the continuous development of deep learning and data science, some new prediction models based on deep learning algorithms have been shown to be more advantageous because their structure can better discover internal laws and external connections between input data and output data. The long short-term memory (LSTM) network is a kind of deep learning neural network that has demonstrated outstanding achievements in many prediction fields. This paper applies the LSTM network directly to the prediction of toxic gas diffusion and uses the Project Prairie Grass dataset to conduct experiments. Compared with the Gaussian diffusion model, support vector machine (SVM) model, and back propagation (BP) network model, the LSTM model of deep learning has higher prediction accuracy (especially for the prediction at the point of high concentration values) while avoiding the occurrence of negative concentration values and overfitting problems found in traditional artificial neural network models.

## 1. Introduction

In recent years, toxic gas leaks caused by chemical plant explosion accidents, forest fires, etc., have frequently occurred in various countries, seriously affecting people’s lives, health, and property. In 2019, chemical plant explosions in Yancheng, China and Houston, USA and forest fires in Sichuan, China all caused a large area of toxic gas leakage and diffusion, which not only harmed people’s health but also greatly hindered rescue work. Therefore, it is very important to predict the diffusion rules of toxic gases and improve the capabilities of disaster response.

The current widely-used gas diffusion models are mainly divided into two categories, one based on mathematical calculations and the other based on historical sample modeling. Typical examples of mathematical calculations include Gaussian diffusion models and computational fluid dynamics (CFD) models [1]. The Gaussian diffusion model uses plain mathematical formulas that can be easily calculated and cost less time, but it only applies to describing unobstructed gas flow over flat terrain and its predictions in complex environments are often unreliable [2]. The CFD model can flexibly represent complex geometries and maintain high accuracy in the calculation of gas diffusion over urban terrain with buildings [3,4,5], so it is widely used for the prediction of toxic gas diffusion in various urban terrains. Nevertheless, there is a big drawback in that it takes too long for CFD to perform necessary calculations, and sometimes even more than several hours. When a sudden accident occurs, it is difficult to guide the rescue in a timely and effective manner using the CFD model, so the capability of emergency response is poor. Driven by artificial intelligence and data science, many researchers have proposed modeling methods based on historical samples (such as neural networks and machine learning) to find the complex relationship between predictive input and output. A new approach that is an integration of gas detectors, artificial neural networks (ANN), and the PHAST model has been proposed [6]. As long as the model is trained in advance, the model parameters can be directly used to predict the gas concentration in this approach. D. Ma and Z. Zhang discussed a series of models of machine learning algorithms and combined the classical Gaussian model with these machine learning algorithms to identify emission source parameters [7]. The results of a model using artificial neural networks coupled with cellular automata designed to calculate the atmospheric dispersion of methane in 2D show that while it maintains acceptable accuracy, its time requirement is far superior to that of CFD [8]. Although the ANN models in these studies above have shown outstanding ability in gas diffusion prediction, there are still some inadequacies, such as inaccurate prediction of high concentration points and negative concentration values. Recently, under the boom of deep learning, many scholars have begun to study predictive models based on deep learning and found that these models can better describe the relationship between data features and have greater superiority in prediction and classification. Jonggeol Na and Kyeongwoo Jeon et al. defined a non-linear surrogate model based on deep learning which employs a variational autoencoder with deep convolutional layers and a deep neural network with batch normalization for the real-time analysis of toxic gas release [9]. The deep belief network (DBN) and the convolutional neural network (CNN) were proposed to construct a new deep learning diffusion model. Compared with traditional machine learning, experiments have proved that the CNN model performs best in terms of accuracy, prediction time, and calculation time [10]. 

In 1997, Sepp Hochreiter and Jürgen Schmidhuber proposed the long short-term memory network (LSTM) as a kind of recurrent neural network [11]. This special deep learning network is widely used for text analysis and time-series data prediction [12,13]. For instance, Google deployed two layers of deep LSTM [14] to build a large-scale speech recognition model, and that model achieved advanced results. A time-weighted LSTM model was put forward to redefine the prediction of stock trends [15], and it outperformed the traditional stock forecasting model. The application of LSTM models in the field of environmental science is also increasing and becoming popular. For instance, they have been used to establish a more effective and robust forecasting model of wind speed [16], to predict hourly day-ahead solar irradiance using weather forecasting data [17], and to monitor carbon dioxide fluxes in forest environments [18]. Although no one has directly applied LSTM to the prediction of toxic gas diffusion, the performance of LSTM or its combination in similar prediction of the coalmine gas concentration [19] and PM_2.5_ [20,21] surpasses that of traditional artificial neural networks. The latest research used sensor data generated by CFD simulation for a real chemical plant to establish a model for real-time prediction of suspected leak locations based on LSTM [22]. According to the principle of toxic gas diffusion, this paper applies a specially designed LSTM model directly to gas diffusion in the real environment. In addition, experiments with multiple models were performed using a classic public dataset to compare the performance of each model.

The organization of the remainder of this paper is as follows. Section 2 introduces the theoretical foundation of this paper, including a brief description of the dataset, LSTM operational principle, and overfitting processing. Section 3 focuses on the implementation process and method of this paper. First is data preprocessing, then the model is designed, and finally, the performance is evaluated. Section 4 analyzes and discusses the experimental results. Finally, conclusions are reached and future work is described in Section 5.

## 2. Theories

### 2.1. Brief Description of Dataset

Project Prairie Grass [23,24] was conducted from July to August 1956, and its dataset remains one of the most comprehensive atmospheric dispersion datasets in field experiments which can reflect the diffusion law of toxic gases. The experimental site was located approximately five miles northeast of O’Neil, Nebraska (42.49 degrees North Latitude and 98.57 degrees West Longitude). During the experiment, the point source was released at a height of 0.46 m above the ground, and SO_2_ was used as a tracer to sample the concentration values every 10 min at a height of 1.5 m along five concentric arcs located 50, 100, 200, 400, and 800 m downwind of the source [25]. The dispersion experiment also involved a great deal of micrometeorological observations and the gathering of a number of data such as air temperature, soil temperature, wind direction, wind speed (seven heights, where the average wind speed is 1 m from the ground), and so on. It is the best choice for our experiment because the Project Prairie Grass dataset can reflect the diffusion rule of toxic gases in a real environment. Due to the experimental requirements, we sorted the dataset to get 68 different versions of the data with each version having multiple observations. In total, there were 8173 valid samples, and some of the common feature parameters are shown in Table 1.

### 2.2. The Long Short-Term Memory Network

**Figure 1:**
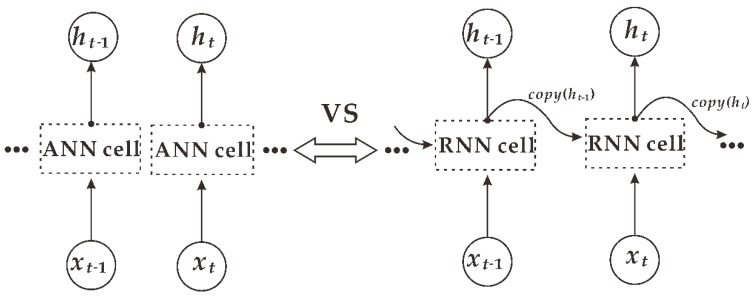
Structural comparison between the artificial neural network (ANN) and recurrent neural network (RNN); ANN on the left is an independent structure, and RNN on the right is an interconnected structure.

The long short-term memory network (LSTM) is a special type of recurrent neural network (RNN). A traditional artificial neural network (ANN) is fully connected between layers and has no connection within the layer, while the hidden layers of the RNN are connected to each other [11]. A contrast of the structures of the ANN and RNN is displayed in Figure 1. The outputs of an ANN are independent of each other; the output of an RNN is not only affected by the current input features but also influenced by the output of the previous moment, so the RNN has better time series performance.

**Figure 2:**
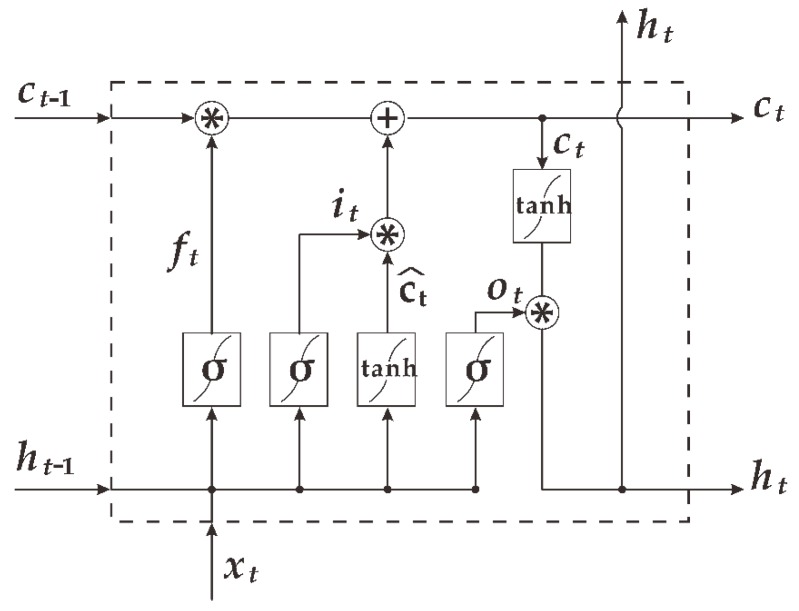
The memory cell structure of the long short-term memory network (LSTM) hidden layer.

In practice, it is difficult to obtain good training of an RNN; the main reason for this is the vanishing gradient and exploding gradient problems described by Bengio et al. [26,27]. Consequently, we are more concerned with its variants, and LSTM is one of them. LSTM and RNN have a similar structure, but the memory cell structure of the hidden layer is different. The LSTM is an integration of a forget gate, input gate, and output gate in the hidden layer’s memory cell which is based on the RNN structure. The forget gate determines how much information should be dropped from the cell. The input gate may pick out which information needs to be updated in the cell. The output gate decides what information will be output from the cell in the end. The design of these three special gate structures effectively solves the problem of the vanishing gradient and has a memory function, which is very appropriate for dealing with long-term dependent problems. Figure 2 shows the memory cell structure of the LSTM hidden layer.

The general working principle of the LSTM can be expressed by Equations (1)–(6):

(1) $$i_{t}=\sigma \left(W_{i}x_{t}+H_{i}h_{t-1}+b_{i}\right)$$

(2) $$f_{t}=\sigma \left(W_{f}x_{f}+H_{f}h_{t-1}+b_{f}\right)$$

(3) $$o_{t}=\sigma \left(W_{o}x_{t}+H_{o}h_{t-1}+b_{o}\right)$$

(4) $${\hat{c}}_{t}=\tanh\left(W_{c}x_{t}+H_{c}h_{t-1}+b_{c}\right)$$

(5) $$c_{t}=f_{t}*c_{t-1}+i_{t}*{\hat{c}}_{t}$$

(6) $$h_{t}=o_{t}*\tanh\left(c_{t}\right)$$

where $f_{t}$, $i_{t}$, and $o_{t}$ denote the forget gate, input gate, and output gate, respectively; σ represents the sigmoid activation function with the range 0 to 1; tanh represents the hyperbolic tangent activation function which outputs values between −1 and 1; ${\hat{c}}_{t}$ is the candidate value for the states of the memory cell at time *t*, and ${\hat{c}}_{t}$ is the state of the current memory cell at time *t*; $h_{t}$ is the output value filtered by the output gate; *W* ( $W_{i}$, $W_{f}$, $W_{o}$, $W_{c}$ ) and *H* ( $H_{i}$, $H_{f}$, $H_{o}$, $H_{c}$ ) are weight matrices; and *b* ( $b_{i}$, $b_{f}$, $b_{o}$, $b_{c}$ ) denotes bias vectors.

### 2.3. Overfitting

**Figure 3:**
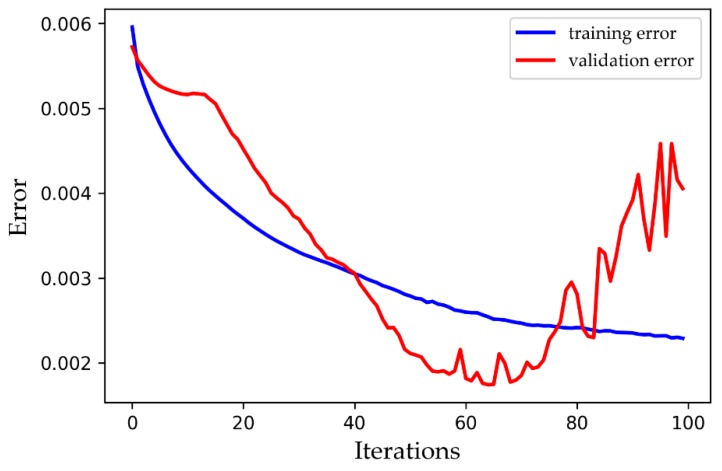
An example of overfitting.

In deep learning, model overfitting is a widespread problem [28]. For instance, by overtraining when the training data are insufficient or the number of features is relatively large, the training effect of the model may be better, but the test or verification effect may be poor. A similar error result with overfitting was observed, as displayed in Figure 3. While the training error decreased with the increasing iterations, the verification error began to increase gradually after decreasing to a certain number of iterations.

**Figure 4:**
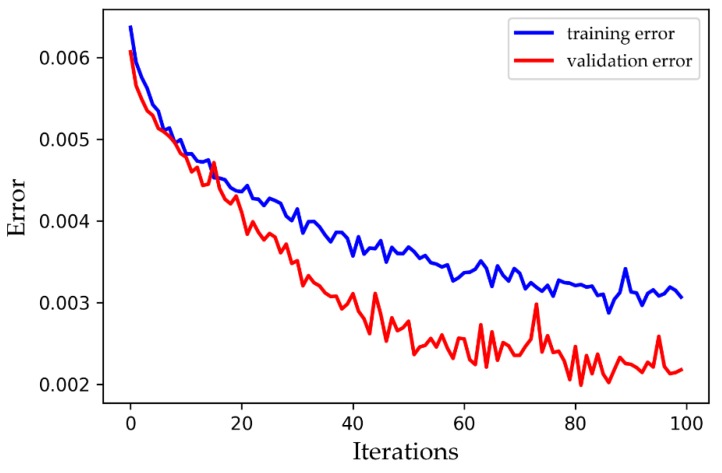
A comparison of the training error and validation error after implementing dropout.

In order to enhance the generalization ability of deep learning models, overfitting must be avoided. The main approaches are L1 and L2 regularization, data augmentation, feature selection, dropout, etc. Regularization is the addition of a constraint to the cost function to reduce some parameters. Data augmentation is a way to increase training samples and expand the dataset. Feature selection means choosing an approach to select the most influential feature and reducing irrelevant features. This paper adopts dropout, one of the most commonly-used methods in deep learning, to prevent overfitting in the neural network. The key idea of dropout is to drop units (along with their connections) from the neural network randomly during training to prevent units from co-adapting too much [29]. Figure 4 shows a comparison of the training error and verification error after implementing dropout, and both are gradually decreasing overall.

## 3. Methods

Most of the experiments were performed in Python on a system with 64-bit Windows, a 4.0 GHz, Intel Core i7-6700K CPU, and 16 GB RAM. However, a small number of experiments were carried out using MATLAB (R2014b, MathWorks, Natick, MA, USA) to compare with the Python results, because most scholars in previous related studies used MATLAB. In the end, we chose to present the results of the Python experiment. The reason for this is that although MATLAB has greater superiority in terms of simulation and calculation speed, it has poor portability and needs to be purchased. Therefore, it is not easy to use for systematic development. In order to make our future work more valuable in practical engineering applications, we chose free, open source, and portable Python to conduct our experiments.

### 3.1. Data Preprocessing

**Figure 5:**
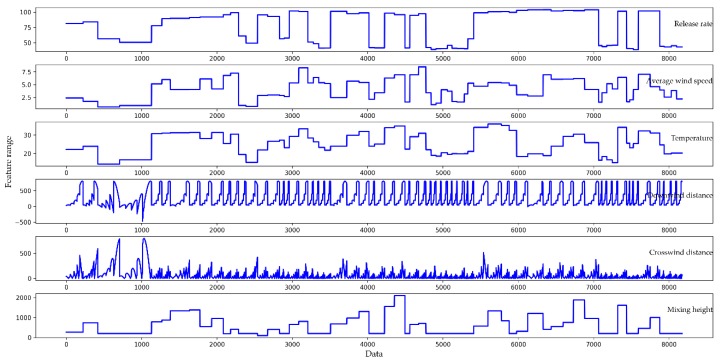
The feature parts of the original dataset: from top to bottom, the representative features are release rate, average wind speed, temperature, downwind distance, crosswind distance, and mixing height.

Original datasets are usually chaotic, and there are inevitably illegal values (a non-float type of datum existing in what should be a float type) and null values, so it is difficult to analyze directly. For this reason, it is necessary to perform data preprocessing, such as culling null values, replacing illegal values, and digitizing eigenvalues. The feature parts of the original dataset are shown in Figure 5. It can be seen that some of the feature values are very large (exceeding 2000), some feature values are very small (close to 0), and there are negative feature values.

**Figure 6:**
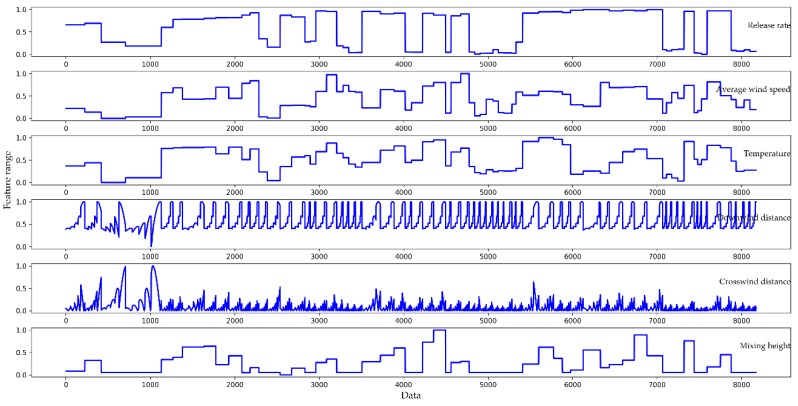
The parts of the normalized features, corresponding to Figure 5.

Normalization operations must be performed on the preprocessed data to avoid attributes in greater numeric ranges dominating those in smaller numeric ranges [30]. In this paper, we used MinMaxScaler normalization [31] which linearly maps all feature values to between 0 and 1. The MinMaxScaler normalization can be expressed as Equation (7):

(7) $$x_{i\_normal}=\frac{x_{i}-\min\{x_{i}\}}{\max\{x_{i}\}-\min\{x_{i}\}}$$

where $x_{i}$ denotes the feature matrix of the *i*th column; $x_{i\_normal}$ denotes the feature matrix after normalization; and $\max\{x_{i}\}$ and $\min\{x_{i}\}$ represent the maximum and minimum values of the current feature matrix, respectively. The parts of the normalized features are shown in Figure 6. In contrast to Figure 5, the trend for each feature in Figure 6 is the same as in the original dataset, but the data range of the feature is reduced to the same interval.

Due to the normalization, the final predicted concentration values are also normalized, so the result must be anti-normalized to restore the original interval. The process of recovery can be expressed as Equation (8):

(8) $$x_{i\_restore}=x_{i\_normal}\cdot (\max\{x_{i}\}-\min\{x_{i}\})+\min\{x_{i}\}$$

The normalized dataset needs to be divided into training sets and testing sets before building the predictive model. Since LSTM is time-correlated, we sort the dataset in chronological order instead of scramble sorting with cross-validation. The first 60 versions of the 7563 data samples were selected as the training set, and the last 8 versions of the 580 sample data were used as the testing set. When the model was trained with the training set and saved, its parameters could be read directly to predict the concentration value during the test.

### 3.2. Model Design

This paper designs an LSTM model with dropout to predict the diffusion of toxic gases in real-world scenarios. The LSTM model consists of three parts: the input layer, the hidden layer, and the output layer. The input layer consists of parameters of all features affecting the gas concentration, and the data were preprocessed (including 20 features such as downwind distance, release rate, average wind speed, temperature, etc.). The neural network here uses a structure with a three-layer hidden layer to achieve higher accuracy of the model and uses dropout, introduced earlier, to prevent overfitting. We selected the Rectified Linear Unit (Relu) function as the activation function between the hidden layers. The Relu function can be expressed as Equation (9), and the value range of the function is greater than 0:

(9) $$f(x)=\{\begin{array}{cc} 0, & x\leq 0\\ x, & x>0 \end{array}$$

**Figure 7:**
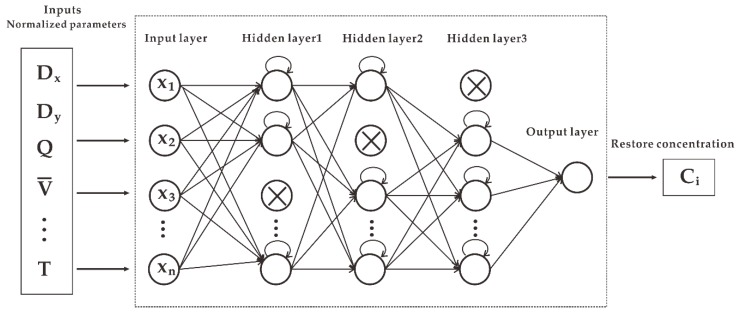
The structure of the LSTM model with dropout for toxic gas diffusion prediction.

Compared with the sigmoid activation function of a traditional artificial neural network, Relu can avoid predicting negative concentration values and is superior to other activation functions in terms of statistical performance and computational cost [32]. There is only one neuron for the output layer, which represents the gas concentration value obtained under the currently input feature values. The activation function of the output layer uses Linear to achieve continuous values of the output concentration. Mean Square Error (MSE) was chosen as the loss function. For the optimizer, we compared the results of SGD, Adam, and RMSprop. Finally, RMSprop was chosen as the best weight optimization of the model. The structure of the LSTM prediction model with dropout is shown in Figure 7.

### 3.3. Performance Criteria

The use of a combination of metrics is often required to assess model performance, including but certainly not limited to one [33]. In this paper, the mean absolute error (MAE), root-mean-square error (RMSE) [34], and correlation coefficient (*r*) were selected to assess the performance of each model. MAE represents the average of the absolute error between the predicted value and the actual value. It avoids the situation where the positive and negative phases cancel each other. RMSE is the square root of the ratio of the square of the deviation between the predicted value and the actual value to the number of observations. It is very sensitive to large or small errors in a set of measurements; therefore, it can reflect the accuracy of the prediction well [35]. The correlation coefficient [36] can well show the linear correlation between the predicted value and the actual value. When *r* is close to 1, the predicted value approaches the actual value, so the model performance is better. The smaller the MAE and RMSE, the better the performance of the model. The calculation principles of these three performance indicators are expressed as Equations (10)–(12):

(10) $$\mathrm{MAE}=\frac{1}{n}\sum_{i=1}^{n}\left|C_{i}-C_{i}^{\prime }\right|$$

(11) $$\mathrm{RMSE}=\sqrt{\frac{1}{n}\sum_{i=1}^{n}{\left(\left|C_{i}-C_{i}^{\prime }\right|\right)}^{2}}$$

(12) $$r=\frac{\mathrm{Cov}{(C}_{i},C_{i}^{\prime })}{\sqrt{\mathrm{Var}(C_{i})\cdot \mathrm{Var}(C_{i}^{\prime })}}$$

where $C_{i}$ denotes the actual concentration and $C_{i}^{\prime }$ represents the predicted concentration. The Var function is used to calculate the variance of a matrix, and the Cov function is applied to calculate the covariance of the two matrices.

## 4. Results and Discussion

In this paper, we established the Gaussian diffusion model, BP model, SVM model, and LSTM model for the divided dataset. The comparative performance evaluation result is displayed in Table 2. 

Although the Gaussian diffusion model only needs to input the feature parameters according to Equations (13) to get the result, which is easy to calculate and understand, the calculated concentration is quite different from the real situation. Therefore, it is not suitable for this situation and is limited by the environment:

(13) $$C(x,y,z,H)=\frac{Q}{2\pi \bar{v}\sigma_{y}\sigma_{z}}\cdot (e^{\left[-{\left(z-H\right)}^{2}/2\sigma_{y}^{2}\right]}+e^{\left[-{\left(z+H\right)}^{2}/2\sigma_{z}^{2}\right]})\cdot e^{\left[-y^{2}/2\sigma_{y}^{2}\right]}$$

**Figure 8:**
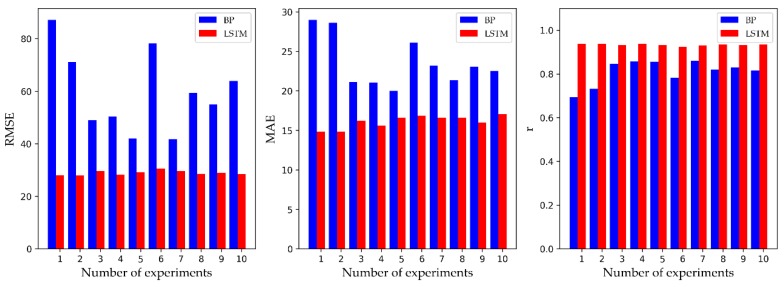
The statistics of 10 experimental results of the BP model and LSTM model. The left-hand side is the RMSE, the middle is the MAE, and the right-hand side is the *r*.

Here, *C* is the concentration of toxic gas at a certain point (*x*, *y*, *z*) in the downwind direction; *x* and *y* denote D*_x_* and D*_y_*, respectively; *z* is the distance from the ground; *Q*, $\bar{v}$, *H*, D*_x_*, and D*_y_* are as described above in Table 1; and $\sigma_{y}$ and $\sigma_{z}$ are the standard deviations that determine the Gaussian distributions in the crosswind and vertical directions [37]. The specific parameter solving process is not explained in detail. Comparing the Gaussian diffusion model with the SVM model, we can see that the *r* and RMSE of the SVM model are better than those of the Gaussian, but the MAE is worse than that of the Gaussian. Overall, the performance of the two models is not much different, and the SVM model has no obvious advantages. The reason for this result may be that the performance of the SVM algorithm itself is very dependent on the choice of parameters. It is difficult to find the best parameters without the help of optimization by an external algorithm. The SVM model and the Gaussian model are difficult to compare in Table 1, but we can see that the BP model and LSTM model are obviously superior in terms of MAE, RMSE, and *r* than the SVM model and the Gaussian model. This is due to the applicability of neural networks to complex nonlinear samples. Although BP has been proven by many studies to have a good effect on the prediction of toxic gas diffusion compared to other commonly used models [6,7], the experimental results in this paper show that the superiority of the LSTM model is obvious. To compare the BP model and LSTM model in greater detail, we recorded the predicted performance of 10 experiments for both models. Figure 8 displays the comparison, and the average corresponds to the data in Table 2. We found that the RMSE and MAE of the LSTM model were smaller than those of the BP model, and *r* was closer to 1. On average, the RMSE and MAE of the LSTM model were 51.63% and 31.72% lower than those of the BP model, and the *r* of the LSTM model was 15.38% higher than that of the BP model. In addition, the results of each experiment of the LSTM model fluctuated little and stabilized at the average. In summary, the LSTM model of this paper is superior to the traditional artificial neural network model in terms of its prediction accuracy and stability. Since the structure of the LSTM model is more complex and adaptable than that of the BP model, it is more capable of portraying the rich intrinsic information of the dataset.

**Figure 9:**
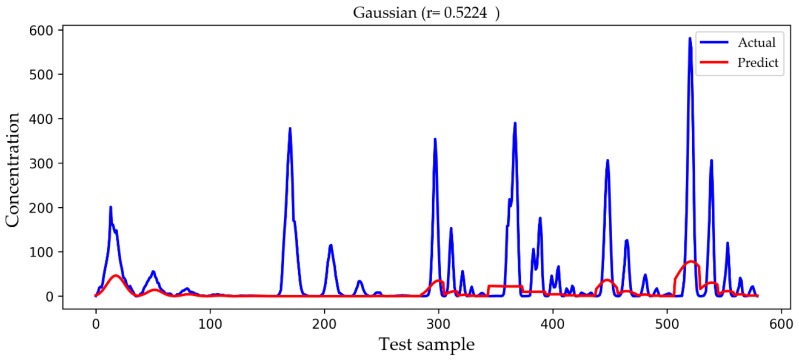
A comparison of the actual and predicted values of the Gaussian diffusion model. Actual drawn in blue represents the true concentration value, and Predict drawn in red represents the predicted result.

**Figure 10:**
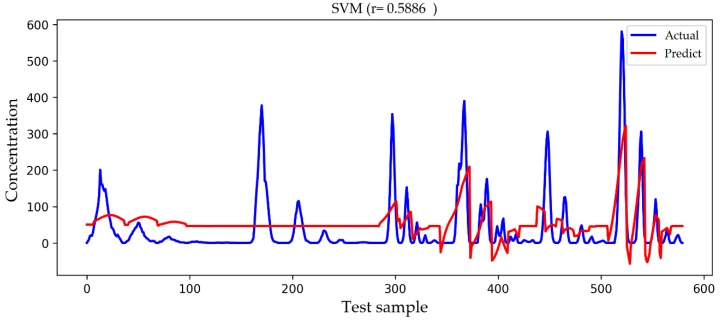
A comparison of the actual and predicted values of the SVM model. Actual drawn in blue represents the true concentration value, and Predict drawn in red represents the predicted result.

**Figure 11:**
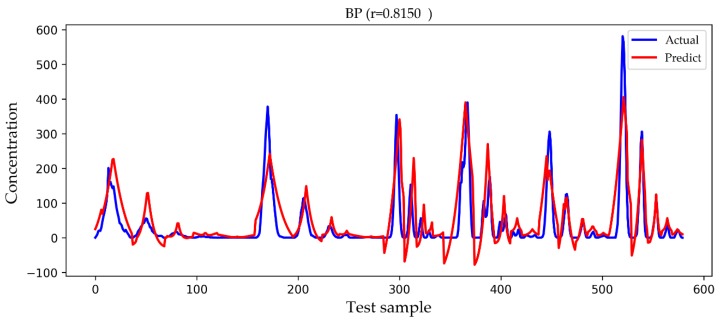
A comparison of the actual and predicted values of the BP model. An independent experiment was taken that was close to the average result but not included in the statistics.

**Figure 12:**
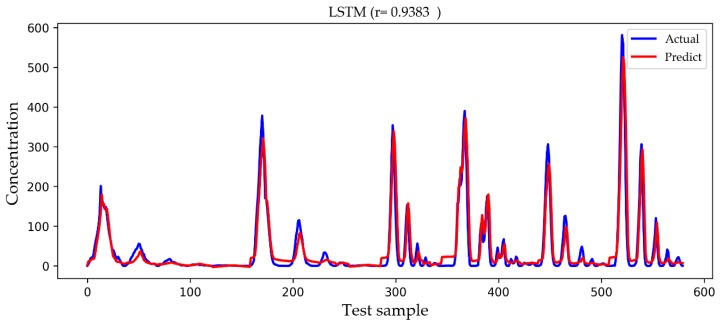
A comparison of the actual and predicted values of the LSTM model. An independent experiment was taken that was close to the average result but not included in the statistics.

In Figure 9, Figure 10, Figure 11 and Figure 12, we show a comparison of the predicted results and actual concentrations of the four models on the testing set, and the testing set has nearly 600 data points. The Gaussian diffusion model and the SVM model are the results of one experiment, corresponding to Table 2. The BP model and the LSTM model show an experiment that is similar to the average results in Table 2. It is shown in Figure 9 that the Gaussian diffusion model approximation reflects the change process of the gas concentration, and there is no negative concentration value, but the prediction is not accurate at the point of high concentration values. In Figure 10, the SVM model is similar to the Gaussian diffusion model, and the high concentration values are also predicted to be inaccurate, but the trend to be fitted has improved. The bad thing is that there are many negative concentration values in the SVM model, which is not in line with the actual situation, so the overall performance of the SVM model does not surpass that of the Gaussian diffusion model. We can see in Figure 11 that the predicted concentration values of the BP model are very close to the actual gas concentration values, and the prediction of the high concentration values is much better than that by the former two models. Unfortunately, negative concentration values that do not conform to the actual situation also appear in the BP model, possibly caused by overfitting. It can be seen from the test results in Figure 12 that the LSTM model designed in this paper not only predicts the high concentration values more accurately but also avoids the occurrence of negative concentration values, so the overall precision is obviously improved. 

We observed the features of several groups of higher concentration values in the testing set and found that they are mostly close to the release source and have a higher release rate, which is in line with the real environment. However, in this paper we do not specifically study how those features affect the concentration value of toxic gases, but we pay more attention to the improvement of the prediction accuracy of the concentration value because accurate prediction of the concentration value of toxic gases is very important for us to judge the hazard level of the current environmental state, which is be a powerful reference for rescuers. It should be noted that different toxic gases have different concentration ranges when determining the hazard level, which is beyond the scope of this paper.

## 5. Conclusions

In this paper, the LSTM deep learning algorithm was applied to the prediction of toxic gas diffusion in a real environment with the aim to make an accurate pre-judgment on the diffusion rule of toxic gases. Compared with the sigmoid activation function in a traditional artificial neural network, the LSTM model of this paper uses the Relu activation function to eliminate negative concentration values and improve the accuracy. Because the training dataset is not big, dropout was used to prevent overfitting and improve the generalization ability of the model. In the experiment, we compared the designed LSTM model with the traditional Gaussian diffusion model, the SVM model of machine learning, and the widely used BP model of a traditional artificial neural network. The experimental results show that the predicted values based on the LSTM deep learning model include no negative concentrations and the prediction of high concentrations is more accurate. Therefore, the LSTM model can better reflect the relationship between features and concentration and is more in line with the actual situation.

The experimental dataset used in this paper is based on a public dataset. Our experiments proved that our model achieved excellent results and provided a good theoretical verification of LSTM for toxic gas diffusion applications. In future work, we are committed to extending the model to an actual designated environment for the purpose of better realizing the practical application value of gas diffusion prediction based on the LSTM model. The initial idea is to use CFD to generate a toxic gas diffusion database in a certain region, use our model to connect to the database, and predict in a timely manner the concentration change distribution map of the region for a period of time. This work can provide timely and effective rescue guidance relating to toxic gas leakage in a certain place.

We hope that through the comparative experiments in this paper, more researchers in the field of environmental science can discover the value of the LSTM model, because it can be applied not only to the prediction of the diffusion of toxic gases but also to general gases (non-toxic gases) or toxic substances (chemical elements).

## Figures and Tables

**Table 1:** A parameter description of the Project Prairie Grass dataset.

Parameters	Symbol	Unit
Downwind distance	D_x_	m
Crosswind distance	D_y_	m
Wind direction	θ	°
Average wind speed	$\bar{v}$	m/s
Version number	No	/
Release rate	Q	g/s
Height of source	H	m
Temperature	T	°C
Height of interest point	Z_o_	m
Mixing height	Z_m_	m
Heat flux	H_f_	W/m^2^
Atmosphere stability length	L	m

**Table 2:** The predictive performance of four different models on the testing set.

Models	RMSE	MAE	*r*
Gaussian	78.6877	34.5548	0.5224
SVM	50.9144	67.0491	0.5886
BP	59.7562	23.5882	0.8093
LSTM	28.9063	16.1069	0.9338

Both the BP model and the LSTM model use the average performance of 10 experiments.
